# Complete Genome Sequences of *Lactobacillus* Phages J-1 and PL-1

**DOI:** 10.1128/genomeA.00998-13

**Published:** 2014-01-02

**Authors:** Maria Eugenia Dieterle, Deborah Jacobs-Sera, Daniel Russell, Graham Hatfull, Mariana Piuri

**Affiliations:** Departamento de Química Biológica, Facultad de Ciencias Exactas y Naturales, Universidad de Buenos Aires, IQUIBICEN-CONICET, Buenos Aires, Argentinaa; Department of Biological Sciences and Pittsburgh Bacteriophage Institute, University of Pittsburgh, Pittsburgh, Pennsylvania, USAb

## Abstract

*Lactobacillus* phages J-1 and PL-1 were isolated during the 1960s from abnormal fermentations of Yakult. The genomes are almost identical, but PL-1 has a deletion in the genetic switch region and also differs in a gene coding for a putative tail protein.

## GENOME ANNOUNCEMENT

*Lactobacillus* phage J-1 was originally isolated in 1965 from an abnormal fermentation of Yakult, a Japanese beverage fermented from skim milk and *Chlorella* extracts, using the *Lactobacillus casei* Shirota strain as a starter (1). *Lactobacillus* phage PL-1 was isolated 2 years later when a strain resistant to J-1 was used for manufacturing this beverage. J-1 and PL-1 are serologically related (2).

Most of the early work on these phages was done by research groups in Japan, including aspects of phage morphology, phage inactivation, adsorption to cells, DNA injection, and characterization of a lytic enzyme (3–10). Although both phages were isolated in the 1960s and were extensively studied for decades, the genome sequences have not yet been available.

Here, we describe the sequencing and genome annotations of phages J-1 and PL-1. The J-1 virion DNA is 40,931 bp and PL-1 is 38,880 bp in length. The G+C% contents are 44.8 and 44.9, respectively. Both phages have unique ends with 10 bases and single-stranded cohesive 3′ extensions (left end, 3′-CGGTCGGCCT).

Using 454 sequencing, phage genomic DNAs were sequenced by the Pittsburgh Bacteriophage Genome Center to a depth of 75-fold coverage for phage J-1 and 45-fold coverage for phage PL-1. Raw reads were assembled using Newbler version 1.1. The assemblies were then quality controlled using Consed version 20. For phage J-1, 14 Sanger reads were required to resolve weak areas in the assembly. No Sanger reads were needed for PL-1. The finished sequences were analyzed and annotated in genome editors, including DNAMaster (http://cobamide2.bio.pitt.edu), GBrowse (11), Glimmer (12), GeneMark (13), tRNAscan-SE (14), Aragorn (15), and then were manually curated. Each of the determined open reading frames (ORFs) was functionally annotated using BLASTp (16), CDD (17), and HHpred (18).

An analysis of the J-1 genome reveals 63 potential ORFs, with 57 genes on the positive strand and 6 genes on the negative strand. The PL-1 genome contains 59 putative ORFs; 57 are rightward transcribed, while two are read in the opposite direction.

Interestingly, the PL-1 and J-1 genomes are almost identical. Compared to J-1, PL-1 has a deletion of 1,900 bp comprising 4 genes that belong to the genetic switch region. PL-1 also differs from J-1 in a gene coding for a putative tail protein (gp16). Since PL-1 was isolated from an abnormal fermentation process using a J-1-resistant strain, gp16 might be involved in host recognition.

The genome organization shares the structure observed in other *Lactobacillus* phages and can be divided into the following modules: packaging, structural proteins, lysis, integration, genetic switch, and replication. An integrase gene (gp24) of the tyrosine recombinase family was found, supporting the temperate origin of these phages.

Regarding the differences found among the J-1 and PL-1 genomes, these data might provide new insights on how phages evolve to counteract bacterial resistance mechanisms.

### Nucleotide sequence accession numbers.

Both genome sequences have been deposited in the GenBank under accession no. KC171646 (J-1) and accession no. KC171647 (PL-1).
